# Motor and cognitive deficits in aged tau knockout mice in two background strains

**DOI:** 10.1186/1750-1326-9-29

**Published:** 2014-08-14

**Authors:** Peng Lei, Scott Ayton, Steve Moon, Qihao Zhang, Irene Volitakis, David I Finkelstein, Ashley I Bush

**Affiliations:** 1 Oxidation Biology Unit, Florey Institute of Neuroscience and Mental Health, The University of Melbourne, Melbourne, Victoria, Australia; 2 Institute of Biomedicine, Jinan University, Guangzhou, Guangdong, China

**Keywords:** Tau, Parkinson’s disease, Alzheimer’s disease, Dementia, Knockout, Aging

## Abstract

**Background:**

We recently reported that Parkinsonian and dementia phenotypes emerge between 7-12 months of age in tau^-/-^ mice on a Bl6/129sv mixed background. These observations were partially replicated by another group using pure Bl6 background tau^-/-^ mice, but notably they did not observe a cognitive phenotype. A third group using Bl6 background tau^-/-^ mice found cognitive impairment at 20-months of age.

**Results:**

To reconcile the observations, here we considered the genetic, dietary and environmental variables in both studies, and performed an extended set of behavioral studies on 12-month old tau^+/+^, tau^+/-^, and tau^-/-^ mice comparing Bl6/129sv to Bl6 backgrounds. We found that tau^-/-^ in both backgrounds exhibited reduced tyrosine hydroxylase-positive nigral neuron and impaired motor function in all assays used, which was ameliorated by oral treatment with L-DOPA, and not confounded by changes in body weight. Tau^-/-^ in the C57BL6/SV129 background exhibited deficits in the Y-maze cognition task, but the mice on the Bl6 background did not.

**Conclusions:**

These results validate our previous report on the neurodegenerative phenotypes of aged tau^-/-^ mice, and show that genetic background may impact the extent of cognitive impairment in these mice. Therefore excessive lowering of tau should be avoided in therapeutic strategies for AD.

## Background

Tau protein is involved in the pathogenesis of Alzheimer’s disease (AD), Parkinson’s disease (PD) and other tauopathies [1]. It is proposed that hyperphosphorylated tau causes neurotoxicity by various mechanisms including tau aggregation and microtubule disassembly [2]. The formation of tau aggregates may interfere with the normal function of tau, by withdrawing the protein from the cytosolic soluble pool [3]. A decrease in soluble tau is observed in affected tissue from AD, PD and other tauopathies [3-5]. Nevertheless, reducing tau protein level has been proposed as a potential therapeutic strategy for AD and epilepsy [6]. Therefore, understanding the normal function of tau protein and the consequences of its reduction is important.

Various functions of tau have been proposed [7], however only a few have been confirmed in tau knockout models. One major proposed function of tau, microtubule stabilization, is likely to have some redundancy from other microtubule-associated proteins such as MAP1B, since tau^-/-^ mice are viable and fertile [8,9]. Tau has also been implicated in axonal transport, but loss of tau does not appear to impact on this cellular function [10-12]. However, loss of tau protein significantly delays the maturation of neurons, and affects the size of the cell body as well as the extent of dendritic arborization in primary cell culture [13,14]. Tau protein also participates in protein trafficking, as loss of tau changes the distribution of several proteins, including Fyn and APP [5,15]. Tau^-/-^ mice display compromised synaptic function, evidenced by impaired long-term potentiation (LTP) at 6-months of age [16], and impaired long-term depression (LTD) at 12-months of age [17] have been reported.

Loss of tau function impacts on mouse behavior. 4-week old tau^-/-^ mice (generated by Harada et al. [8]) have been reported to exhibit motor deficits and muscle weakness [18], and at 3-6 months of age tau^-/-^ mice (generated by Dawson et al. [13], on a Bl6 background) showed deficits in the open field and balance beam tests [19]. Recently, Ahmed et al. [16] reported that tau^-/-^ mice (generated by Tucker et al. [20]) at 6-months of age showed impaired contextual and cued fear conditioning [16]. We previously reported that no significant neurological deficits were observed in tau^-/-^ mice on a Bl6/129sv mixed background (also created by Dawson et al. [13]) prior to 7 months of age, but that they subsequently expressed significant motor and cognitive deficits by 12-months of age, which were caused by toxic brain iron accumulation and associated disruption of dopaminergic pathways [5]. The motor deficits were also observed by two independent groups using the same strain on a Bl6 background at 12-months of age [21,22], but the latter proposed that these deficits might be caused by weight gain [22]. Both reports found no cognitive deficits at this stage, but Ma et al. [21] reported impaired Morris Water Maze performance in tau^-/-^ mice at 20-months of age [21].

Here, we attempt to explain these discrepant results. We considered the environmental and genetic variants in both studies, and performed an extended set of behavioral experiments on 12-months old tau^+/+^, tau^+/-^, and tau^-/-^ mice comparing Bl6 background to Bl6/129sv background. We conclude that the motor dysfunction observed by us and by others is not dependent on mouse weight, environmental or genetic background, however cognitive impairment is dependent on genetic background.

## Results

**Figure 1:**
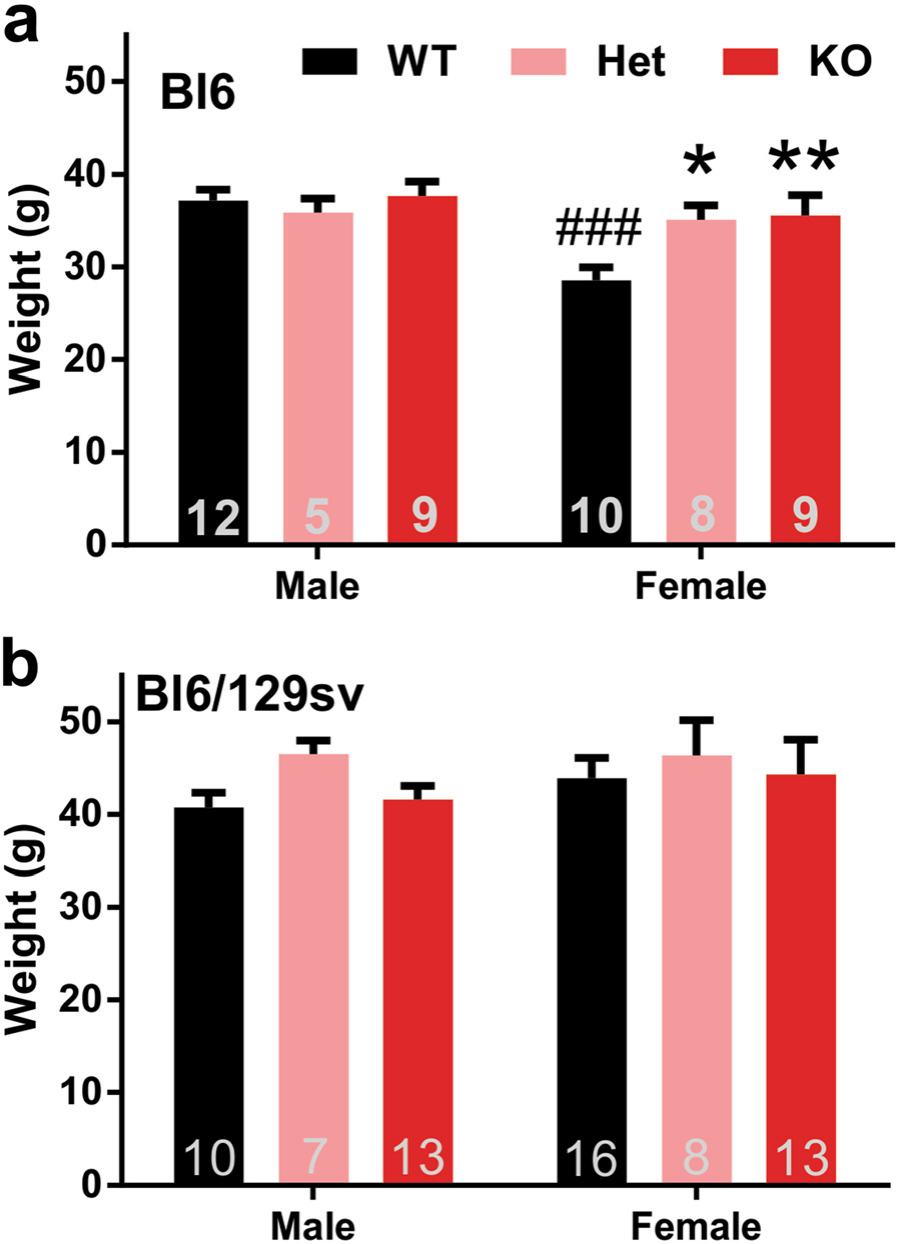
**Body weight in 12-months old Bl6 background and Bl6/129sv background mice. a)** For Bl6 background mice: Two-way ANOVA: genotype (*p* = 0.049), gender (*p* = 0.008) effects and interaction (*p* = 0.038). **b)** For Bl6/129sv background mice: Two-way ANOVA: no genotype (*p* = 0.342), gender (*p* = 0.402) effects, nor interaction (*p* = 0.835). n is indicated in the column of each group. *p < 0.05, **p < 0.01, versus age-matched wild type mice (Bonferroni post hoc test). Data are means ± SEM.

We found no difference in weight between tau^+/+^, tau^+/-^ and tau^-/-^ mice, on the Bl6/129sv background (Figure 1). The female, but not the male, tau^-/-^ and tau^+/-^mice were significantly (≈20%; *p* = 0.005) heavier than female wild type mice on the Bl6 background (Figure 1), similar to the observation by Morris et al. [22]. Unlike the Bl6/129sv strain, female wild type Bl6 mice at 12 months of age were significantly lighter than male mice (*p* < 0.001, Figure 1), a difference that was abolished by tau ablation.

**Figure 2:**
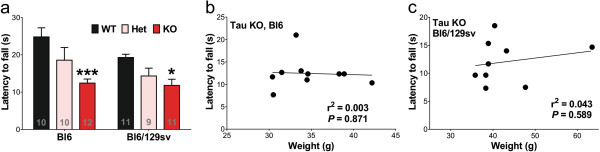
**Complete tau reduction impairs the performance of mice in accelerated Rotarod test at 12 months of age. a)** Tau^-/-^ mice significantly maintained less time on the rod. Two-way ANOVA: genotype effects (*p* = 0.0002) but no genetic background (*p* = 0.061) effects or interaction (*p* = 0.519). * *p* < 0.05, *** *p* < 0.001, versus age-matched wild type mice (Bonferroni post hoc test). n is indicated in the column of each group. Gender distribution is given in Additional file 6: Table S1. Data are means ± SEM. **b-c)** No correlation between weight and Rotarod performance in 12-month-old mice (n = 9 – 11). **b)** Between tau^-/-^ mice in Bl6 background and weight (r^2^ = 0.003, *p* = 0.871 by Pearson’s regression). **c)** Between tau^-/-^ mice in Bl6/129sv background and weight (r^2^ = 0.043, *p* = 0.589 by Pearson’s regression).

We tested the effects of Bl6 background compared to Bl6/129sv background on the motor performance of the tau^-/-^ mice at 12 months of age. We found that the latency to fall in the accelerated Rotarod test was significantly reduced in tau^-/-^ mice in both of genetic backgrounds by the same proportion (-50% for Bl6 background, *p* = 0.003; -43% for Bl6/129sv background, *p* = 0.033, Figure 2a). There was a trend for poorer Rotarod performance in the tau^+/-^ mice in both backgrounds (One-way ANOVA with *post hoc* test for linear trend, for Bl6, *p* = 0.002; for Bl6/129sv, *p* = 0.002).

It was previously reported that latency to fall inversely correlates with mouse body weight, and thus the motor deficits observed in tau^-/-^ mice might be due to increased weight of the mutant [22]. We found no association of weight with latency to fall in either tau^-/-^ mice on Bl6 background (r^2^ = 0.003, *p* = 0.871, Figure 2b), tau^-/-^ mice on Bl6/129sv background (r^2^ = 0.043, *p* = 0.589, Figure 2c), all Bl6 mice (r^2^ = 0.041, *p* = 0.321, Additional file 1: Figure S1a), or all Bl6/129sv mice (r^2^ = 0.087, *p* = 0.114, Additional file 1: Figure S1b). Therefore, the tau^-/-^ mice deficits we observed in the Rotarod test are independent of genetic background or mouse body weight.

**Figure 3:**
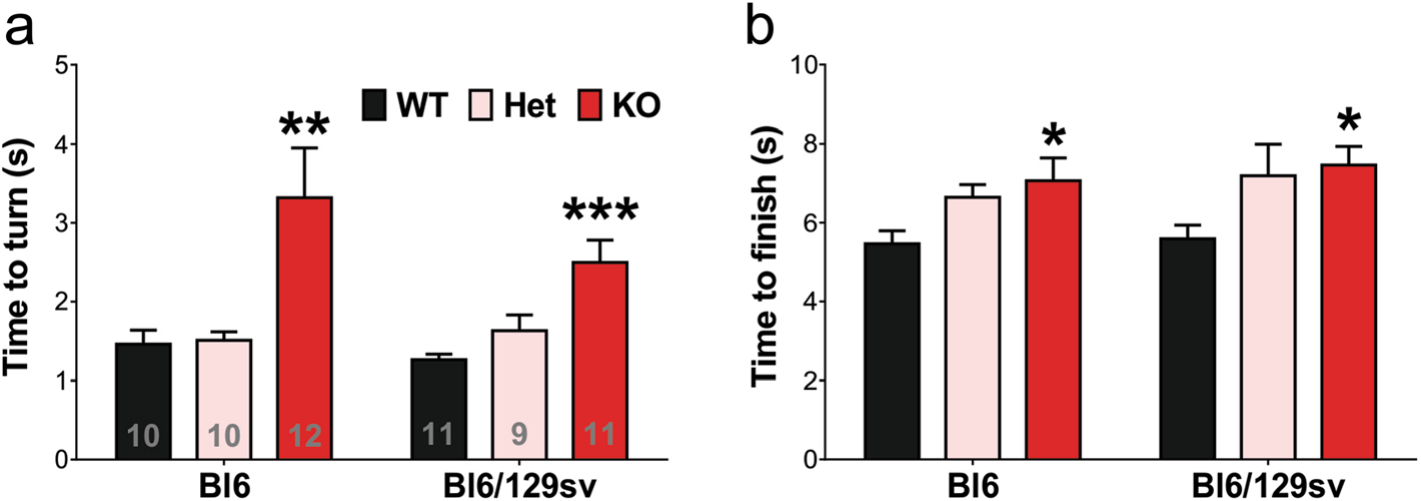
**Complete tau reduction impairs the performance of mice in Pole test at 12 months of age. a)** Tau^-/-^ mice took significantly longer time to turn on the pole. Two-way ANOVA: genotype effects (*p* < 0.0001) but no genetic background (*p* = 0.255) effects or interaction (*p* = 0.352). **b)** Tau^-/-^ mice took significantly longer time to descend the pole. Two-way ANOVA: genotype effects (*p* = 0.001) but no genetic background (*p* = 0.377) effects or interaction (*p* = 0.903). * *p* < 0.05, ** *p* < 0.01, *** *p* < 0.001, versus age-matched wild type mice (Bonferroni post hoc test). n is indicated in the column of each group. Gender distribution is given in Additional file 6: Table S1. Data are means ± SEM.

The Pole test is an established assay for mouse movement dysfunction [23]. We previously reported that tau^-/-^ mice on a Bl6/129sv background took significantly longer than age-matched wild-type mice to both *turn* and *complete* the test [5], a deficit that was also identified by other investigators studying tau^-/-^ mice on a Bl6 background [22]. To directly compare the impact of genetic background, we repeated the study with both knockout and heterozygote strains on both backgrounds, compared to their parental wild-types. This showed that the motor impairments (Time to turn, Time to finish) in the tau^-/-^ strains in the pole test were significant and comparable in either genetic background (Time to turn: for Bl6 background, +127%, *p* = 0.004; for Bl6/129sv background, +97%, *p* < 0.001; Time to finish: for Bl6 background, +29%, *p* = 0.032; for Bl6/129sv background, +33%, *p* = 0.034; Figure 3). The heterozygotes did not express significant deficits but there was a trend to increase in the time to finish parameter compared to background-matched controls (One-way ANOVA with *post hoc* test for linear trend, for Bl6, *p* = 0.017; for Bl6/129sv, *p* = 0.019). There was no correlation between the mouse body weight and time to turn in the Pole test, similar to the results published by Morris et al. [22] (For all Bl6 mice, r^2^ = 0.087, *p* = 0.135; For all Bl6/129sv mice, r^2^ = 0.015, *p* = 0.569; Additional file 2: Figure S2).

**Figure 4:**
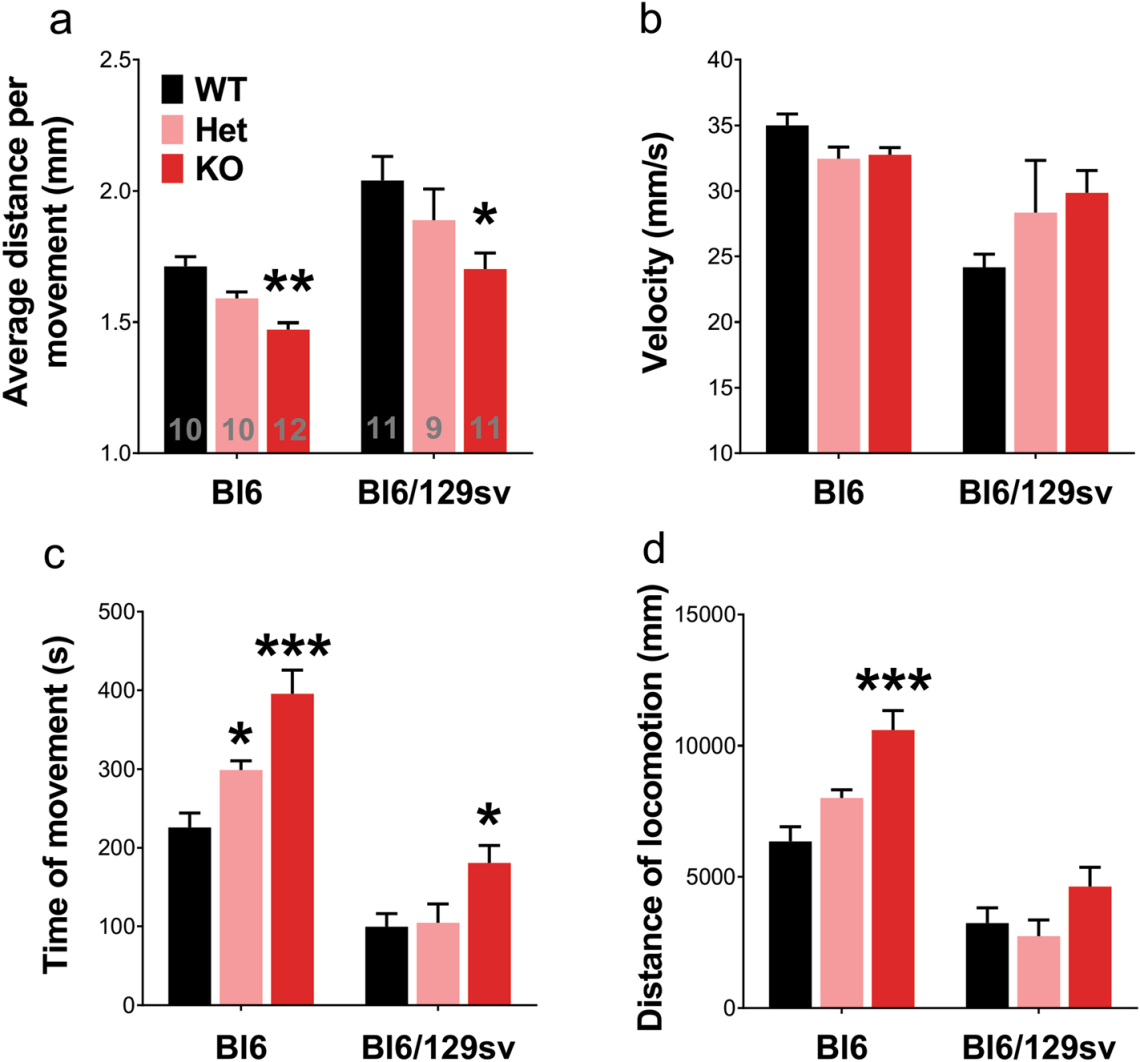
**Complete tau reduction impairs the performance of mice in Openfield test at 12 months of age. a)** Tau^-/-^ mice averagely moved significantly less distance per movement. Two-way ANOVA: genotype (*p* = 0.0002) and genetic background (*p* < 0.0001) effects but no interaction (*p* = 0.725). **b)** Velocity. Two-way ANOVA: genetic background effects (*p* = 0.0003), but no genotype effects (*p* = 0.651) or interaction (*p* = 0.082). **c)** Time of movement. Two-way ANOVA: genotype (*p* < 0.0001) and genetic background (*p* < 0.0001) effects, but no interaction (*p* = 0.121). **d)** Distance of locomotion. Two-way ANOVA: genotype (*p* < 0.0001) and genetic background (*p* < 0.0001) effects, but no interaction (*p* = 0.064). *p < 0.05, ** *p* < 0.01, *** *p* < 0.001, versus age-matched wild type mice (Bonferroni post hoc test). n is indicated in the column of each group. Gender distribution is given in Additional file 6: Table S1. Data are means ± SEM.

We re-assessed tau^-/-^ mice in the Open Field test (the temporal profile data at 5 minute intervals are shown in Additional file 3: Figure S3). Consistent with our previous results [5], we found a significant reduction in the average distance per movement, in both genetic backgrounds (Bl6, -14%, *p* = 0.005; Bl6/129sv, -17%, *p* = 0.014, Figure 4a). The velocity of the tau^-/-^ mice was not affected (Figure 4b), consistent with an increased time in movement (Bl6, +75%, *p* < 0.001; Bl6/129sv, +81%, *p* = 0.031, Figure 4c), and increased distance of locomotion (Bl6, +67%, *p* < 0.001; Bl6/129sv, +43%, *p* = 0.221, Figure 4d). Analysis of the movement of the mice in the first 10 mins revealed similar results to analysis of the full 60 mins (Additional file 4: Figure S4).

**Figure 5:**
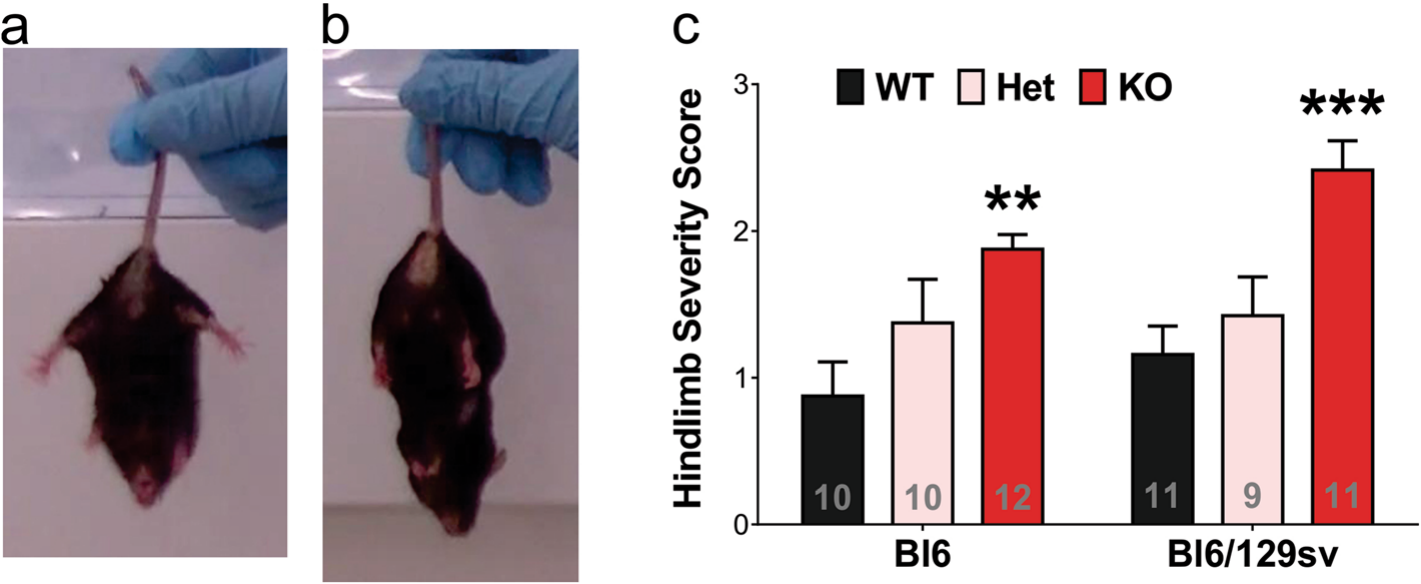
**Altered hindlimb clasping behavior in tau**^**-/- **^**mice at 12 months of age. a-b)** Example of lack of hindlimb clasping (hindlimbs splayed outwards away from abdomen, Scored 0, **a**; and hindlimb clasping behavior towards the abdomen, Scored 3, **b)**. **c)** Tau^-/-^ mice exhibited significantly increased hindlimb clasp severity compared to its background control. Two-way ANOVA: genotype effects (*p* < 0.0001) but no genetic background (*p* = 0.115) effects or interaction (*p* = 0.556). **p < 0.01, ***p < 0.001, versus age-matched wild type mice (Bonferroni post hoc test). n is indicated in the column of each group. Gender distribution is given in Additional file 6: Table S1. Data are means ± SEM.

The hindlimb clasping severity score has been used to characterize mouse models of Parkinson’s disease and other basal ganglia disorders [24-26]. We found a significant difference between both strains of tau^-/-^ mice and their background controls, where tau^-/-^ mice were more prone to clasp (Bl6, +114%, *p* = 0.008; Bl6/129sv, +109%, *p* < 0.001, Figure 5). Tau^+/-^ mice did not express significantly greater clasping but there was a trend compared to wild type mice (One-way ANOVA with *post hoc* test for linear trend, for Bl6, *p* = 0.004; for Bl6/129sv, *p* = 0.001).

**Figure 6:**
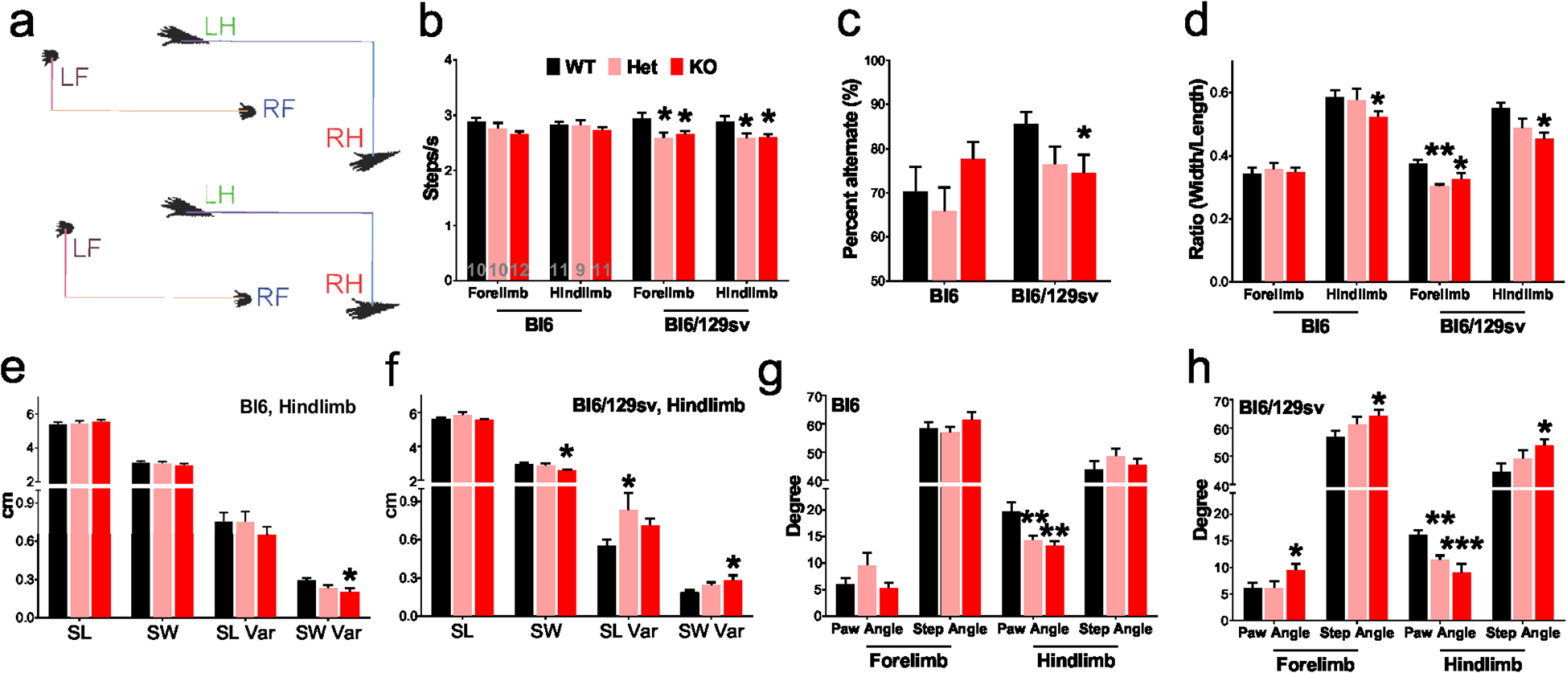
**Altered movement pattern of tau**^**-/- **^**mice at 12 months of age in Digigait analysis. a)** Examples of footprint recorded by the Digigait apparatus. **b-h)** parameters from Digigait analysis: stride frequency **(b)**, percentage alternate gait where perfectly antiphase stepping is 100% alternate **(c)**, stride length normalized to stance width **(d)**, stride length, stance width, and variability of hindlimb stride of mice in Bl6 background **(e)**, stride length, stance width, and variability of hindlimb stride of mice in Bl6/129sv background **(f)**, paw angle and step angle of mice in Bl6 background **(g)**, and paw angle and step angle of mice in Bl6 background **(h)**. Additional gait analysis can be found in Additional file 5: Figure S5. One-way ANOVA were performed to determine the differences versus age-matched wild type mice for each individual parameter. *p < 0.05, **p < 0.01, ***p < 0.001. n is indicated in the column of each group. Gender distribution is given in Additional file 6: Table S1. Data are means ± SEM.

Automated gait analysis using the DigiGait treadmill apparatus revealed clear alterations in the running pattern between tau^-/-^ mice and their background controls (Figure 6, Additional file 5: Figure S5). This test was previously used to characterize 6-OHDA-intoxicated, MPTP-intoxicated, and transgenic PD models [27-29]. The dimensions of the strides (length and width) were not significantly different (Additional file 5: Figure S5a), consistent with their unchanged weight (Figure 1). At a speed of 15 cm/s, we found that tau^+/-^ and tau^-/-^ mice with Bl6/129sv background showed significantly reduced stride frequency for both forelimbs (-12%, *p* = 0.014 for tau^+/-^, and -10%, *p* = 0.047 for tau^-/-^) and hindlimbs (-10%, *p* = 0.026 for tau^+/-^, and -10%, *p* = 0.040 for tau^-/-^, Figure 6b), compared to wild type mice. These tau^-/-^ mice also showed significantly reduced consistency of their running pattern (-11%, *p* = 0.033, Figure 6c). Tau^-/-^ mice on either background showed a significantly reduced ratio between stride length and stance width in their hindlimbs (Bl6, -11%, *p* = 0.049; Bl6/129sv, -17%, *p* = 0.014, Figure 6d), without a major difference in their stride length (Figure 6e-f). Tau^-/-^ mice on the Bl6/129sv background, but not on the Bl6 background, showed reduced stance width (-13%, *p* = 0.017) with increased variance (+5%, *p* = 0.037, Figure 6f), indicating impaired base of support, which is consistent with the results from Rotarod test.

Tau^-/-^ mice on either background also showed significantly altered paw and/or step angles during movement (Figure 6g-h), which are also indications of dysfunctional ataxia [30]. Analysis of other gait dynamics also revealed differences between genotypes, for example, tau^-/-^ mice in both backgrounds showed significant differences compared to wild-type mice for swing, brake, propulsion and stance durations of forelimbs (Additional file 5: Figure S5).

**Figure 7:**
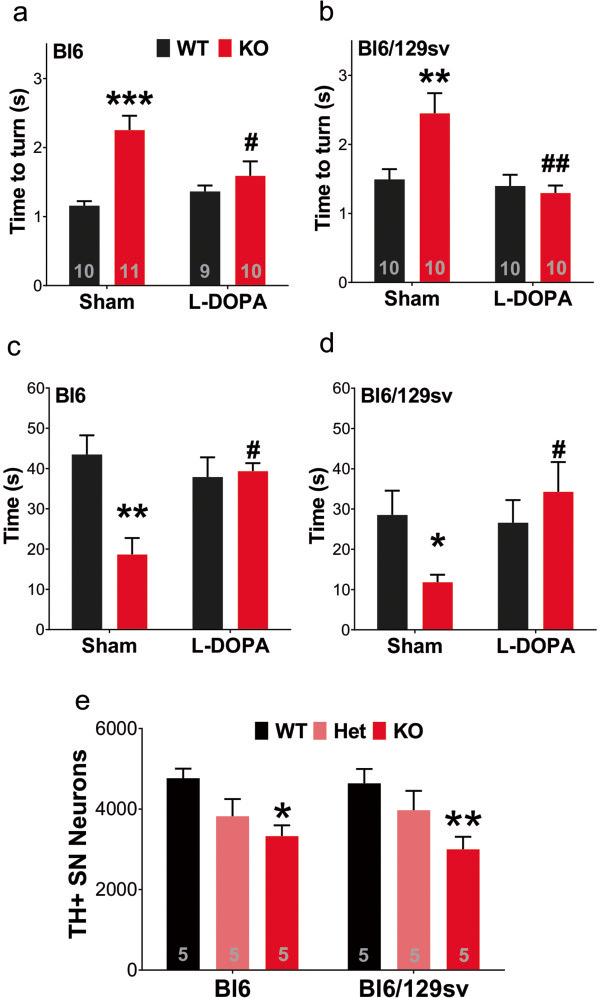
**Motor deficits are related to nigral degeneration. a-d)** Single dose of L-DOPA (10 mg/kg, gavage) rescued motor impairment in 12-month-old tau^-/-^ mice. **a)** Pole test for Bl6 background mice (time to turn). Two-way ANOVA: genotype effects (*p* = 0.0001) and interaction (*p* = 0.008), but no treatment effects (*p* = 0.142). **b)** Pole test for Bl6/129sv background mice (time to turn). Two-way ANOVA: genotype (*p* = 0.032) and treatment (*p* = 0.002) effects, with interaction (*p* = 0.009). **c)** Rotarod test for Bl6 background mice (latency to fall). Two-way ANOVA: genotype effects (*p* = 0.009) and interaction (*p* = 0.041), but no treatment effects (*p* = 0.398). **d)** Rotarod test for Bl6/129sv background mice (latency to fall). Two-way ANOVA: genotype effects (*p* = 0.032) and interaction (*p* = 0.047), but no treatment effects (*p* = 0.698). *p < 0.05, **p < 0.01, ***p < 0.001, versus age-matched wild type mice (Bonferroni post hoc test). *#* < 0.05, ## *p* < 0.01, versus sham-treated tau^-/-^ mice (Bonferroni post hoc test). **e)** 12-month-old tau^-/-^ mice showed reduced TH-positive nigral neuron number. Two-way ANOVA: genotype effects (*p* = 0.001), but no genetic background (*p* = 0.729) effects or interaction (*p* = 0.799). * *p* < 0.05, **p < 0.01, versus age-matched wild type mice (Bonferroni post hoc test). n is indicated in the column of each group. Gender distribution is given in Additional file 6: Table S1. Data are means ± SEM.

We showed previously that L-DOPA gavage rescued the motor dysfunction observed in tau^-/-^ mice [5], at variance with the findings of Morris et al. [22]. Those authors reported that the motor deficits on the Pole Test observed in tau^-/-^ mice on a Bl6 background were not rescued by single I.P. or chronic L-DOPA therapy, compared to sham-treated controls [22]. We believed it important to note, though, that the results of Morris et al. [22] show that the tau^-/-^ mice on a Bl6 background expressed a significant impairment in the Pole Test before the L-DOPA treatment, which was abolished after the treatment, indicating that the treatment might have indeed partially rescued the phenotype. Therefore, we repeated the experiment on tau^-/-^ mice of both genetic backgrounds, and found, in our hands, that L-DOPA rescued the Pole test and Rotarod disability in both strains. This independent cohort of 12-months old tau^-/-^ mice (in both genetic backgrounds) showed once again significantly increased time to turn in Pole Test (+95%, Bl6, *p* < 0.001; +64%, Bl6/129sv, *p* = 0.009, Figure 7a-b), and significantly decreased latency to fall in the Rotarod test (-58%, Bl6, *p* = 0.005; -59%, Bl6/129sv, *p* = 0.049, Figure 7c-d). A single oral dose of L-DOPA completely corrected the motor behaviors to similar levels as their background controls one hour after treatment (Pole test, -42%, Bl6, *p* = 0.033; -90%, Bl6/129sv, *p* = 0.001; Rotarod, +111%, Bl6, *p* = 0.049; +190%, Bl6/129sv, *p* = 0.044, compared to sham-treated tau^-/-^ mice), indicating that the behavioral abnormality observed in tau^-/-^ mice involves dysfunction of dopamine-related pathways, consistent with nigral neurodegeneration we previously described [5].

To further assess the nigral neurodegeneration observed, we stereologically counted the tyrosine hydroxylase-positive neurons in the substantia nigra pars compacta. Consistent with the motor dysfunction, loss of tau protein induced nigral neuron loss independent of genetic background (-30%, Bl6, *p* = 0.0181; -36%, Bl6/129sv, *p* = 0.007, Figure 7e). This is consistent with our previous report [5]. Tau^+/-^ mice in both backgrounds exhibited a trend to nigral neuron loss (One-way ANOVA with *post hoc* test for linear trend; Bl6 *p* = 0.0172; Bl6/129sv *p* = 0.009, Figure 7e).

**Figure 8:**
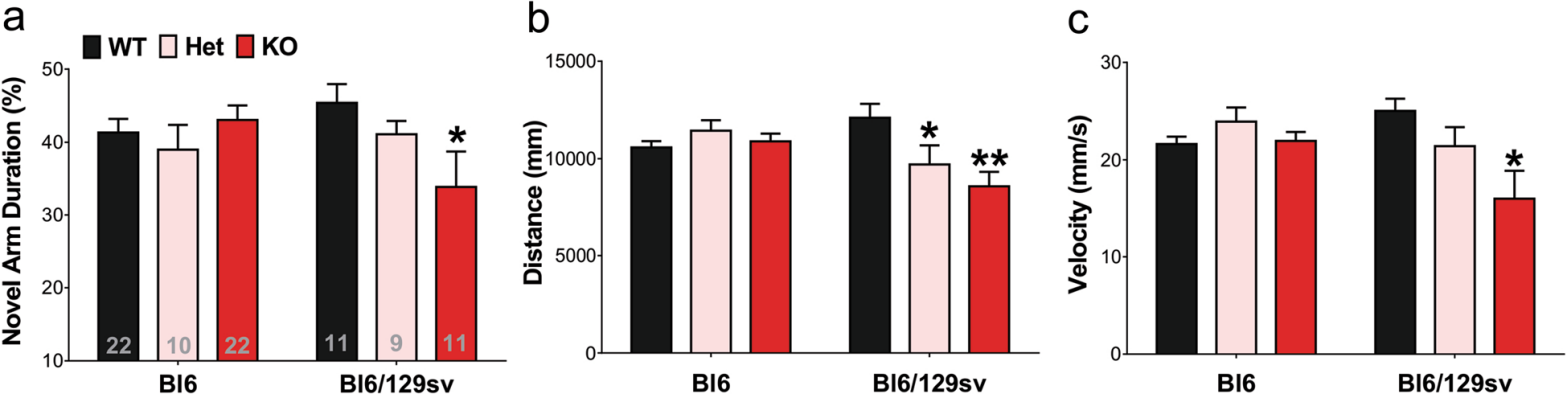
**Genetic background varies behaviors of mice at 12-months of age in Y maze test. a)** Novel arm duration time (% time spent in novel arm during 5 mins recording time) of Y maze test. Two-way ANOVA: interaction (*p* = 0.04), but no genetic background (*p* = 0.664), or genotype (*p* = 0.182) effects. Additional one-way ANOVA were performed to determine if genotype accounts for variance in Bl6/129sv group. **b)** Total distance of movement during the test. Two-way ANOVA: interaction (*p* = 0.002) and genotype effect (*p* = 0.025), but no genetic background effect (*p* = 0.093). **c)** Velocity of movement during the test. Two-way ANOVA: interaction (*p* = 0.003) and genotype effect (*p* = 0.005), but no genetic background effect (*p* = 0.157). *p < 0.05, **p < 0.01, versus age-matched wild type mice (Bonferroni post hoc test). n is indicated in the column of each group. Gender distribution is given in Additional file 6: Table S1. Data are means ± SEM.

We interrogated the cognitive function of 12-month old tau^-/-^ mice using the Y-maze test. Consistent with our previous observation [5], tau ablation in the Bl6/129sv background caused a significantly decreased time spent in the novel arm compared to wild-type controls (*p* = 0.042, Figure 8a), indicating a spatial memory deficit. Tau^+/-^ mice in the Bl6/129sv background also exhibited a trend of Y maze impairment (One-way ANOVA with *post hoc* test for linear trend, *p* = 0.017, Figure 8a). But in the Bl6 background, tau^-/-^ mice showed no difference in time spent in the novel arm compared to tau^+/+^ mice, indicating normal spatial memory (Figure 8a). Thus, the Bl6/129sv background engenders greater vulnerability for cognitive degeneration caused by loss of tau. Additionally, we found that tau^-/-^ mice in the Bl6/129sv background showed reduced distance of locomotion (-30%, *p* = 0.002, Figure 8b), as well as reduced velocity (-36%, *p* < 0.001, Figure 8c) in the same Y maze test, in accord with their observed motor deficits. Tau^+/-^ mice in the Bl6/129sv background also exhibited reduced distance of locomotion (-20%, *p* = 0.023, Figure 8b) and a trend to slower velocity (One-way ANOVA with *post hoc* test for linear trend, *p* = 0.003, Figure 8c). The tau^-/-^ mice in the Bl6 background ran a similar distance compared to wild type mice (Figure 8b) and at a similar speed (Figure 8c).

We considered whether dietary iron exposure, as a variable between laboratories, could contribute to the toxic accumulation of brain iron in the tau^-/-^, which we had reported drives the motor and cognitive degenerative phenotypes [5]. The dietary iron content in the food in our current study was markedly less than in our previous study (78 ppm vs 590 ppm, Table 1), and compared with the chow used by Morris et al. (78 ppm VS 220 ppm) [22]. Therefore, dietary iron exposure is unlikely to account for the emergence of the phenotype.

**Table 1:** Elementary components of mouse chow used in Melbourne Brain Center

	**μg/g**	**S.E.**
Aluminum	40.26	14.479
Manganese	88.93	10.561
Iron	78.09	7.237
Cobalt	0.51	0.096
Copper	9.91	4.158
Zinc	50.96	3.457
Selenium	0.32	0.028
Molybdenum	1.14	0.477
Cadmium	0.02	0.004

## Discussion

### Evidence of PD-like contextual kinetic abnormalities in tau^-/-^ mice

The current study replicates the findings we published previously [5], and partially reconciles some of the findings that were at variance with those of Morris and colleagues [22]. In particular, we found that introduction of the C57BL/6 strain into the original F1 FVB/129 background [13] did not alter the Parkinson-like motor deficits that we originally described [5]. We consistently observe a L-DOPA responsive motor impairment in tau^-/-^ mice independent of the mouse body weight and genetic background, which we interpret as a PD-like basal ganglia dysfunction phenotype. This is further supported by our consistent observation of TH-positive nigral neuron loss. Others have also reported reduction in TH expression in the substantia nigra in tau^-/-^ mice [21], similar to our observations but inconsistent with findings of Morris et al. [22]. Tau is implicated in PD by being one of the major genetic risk factors [31-34], and by interacting with α-synuclein [35-39]. There is a marked loss of soluble tau in nigral tissue from PD cases [5]. Our current data confirms that loss of normal soluble tau could contribute to the motor deficits of PD.

We found in the current study that tau^-/-^ mice on two different genetic backgrounds showed significant reduction in average distance per movement in Open Field Test (Figure 4a), but increased time in movement and increased (Bl6 only) distance of locomotion (Figure 4c,d). Their velocity remained unchanged (Figure 4b). We interpret this as hyperkinesis with dyskinetic movements. The tau^-/-^ mice spent more time moving, but took smaller steps than normal mice. This is consistent with the animal being unable to suppress movement, when there is lack of cognitive intention (in contrast to the Y-maze task, *vide infra*), as seen in PD. In addition, the increase in anxiety of the Open Field Test could also be releasing more movements in the tau^-/-^ mice. This would also be a phenocopy of PD where anxiety exacerbates motor symptoms and disability [40]. In contrast, motor data from the Y-maze Test showed that tau^-/-^ mice in a Bl6/129sv background had a reduced total distance of movement and velocity (Figure 8b,c). One hour Open Field Test incorporates anxiety cues (prefrontal and amygdala), whereas the 5 mins Y-maze Test involves more frontal lobe and cognitive input, where the impaired memory of the tau^-/-^ mice (Figure 8a) could limit the drive to explore.

### Variance induced by genetic background

Our current findings also show that genetic strain may contribute to the cognitive degeneration we reported previously for tau^-/-^ mice in a Bl6/129sv background [5], as tau^-/-^ mice in the Bl6 background did not express this difference compared with wild type Bl6 mice in the Y-maze test. Morris et al. additionally performed Morris water maze and novel object recognition tests of tau^-/-^ mice in the Bl6 background, and found no cognitive behavioral deficits [22]. However, Ma et al. reported that tau^-/-^ mice in the Bl6 background showed deficits in Morris Water Maze test at 20-months of age [21], therefore it is possible that the velocity of cognitive degeneration in tau^-/-^ mice is dependent on genetic background.

### Variance induced by environmental and dietary factors

The phenotype of tau^-/-^ mice may also be sensitive to environmental and dietary factors. In our previous study, the mice were housed in a conventional animal facility [5], in contrast to the specific pathogen free (SPF) facility used by Morris et al. [22]. In the current study, we re-derived our colony in a SPF facility. Since the tau^-/-^ mice in our SPF facility showed similar deficits to the same strain in our previous conventional facility, we conclude that the presence of infectious pathogens in the facility is unlikely to account for the phenotype we observe.

While we found no evidence that dietary iron levels could account for the differences in phenotypes between laboratories, we noticed that the mouse chow used by Morris et al. contained 0.33% Omega-3 Fatty Acids, compared to 0.07% in our previous mouse chow, and 0% in our current chow. This raises the possibility that Omega-3 Fatty Acids in the diet, which are known to affect neurodegeneration [41,42], could offset the effects of tau loss on age-related iron accumulation and subsequent toxicity. In fact, Ma et al. reported in their recent publication that dietary supplementation with docosahexaenoic acid (DHA) rescued both motor and cognitive deficits they observed in tau^-/-^ mice in Bl6 background [21], suggesting that dietary differences may account for some variance.

### Possible variations induced by aging

One recent study using Bl6 background mice also reported that they could not see differences in motor phenotype or brain iron levels between tau^-/-^ and WT mice at 24 months of age [43], which is at apparent variance with findings of ours [5] or Ma et al. [21]. As we previously suggested, the deficits in the tau^-/-^ mice represent a form of accelerated aging where the mouse manifests brain iron accumulation that is different from age-matched controls from 7 to 12 months. Brain iron levels in normal mice invariably rise with age [44-46], and accompany cognitive and motor deficits, but the elevated iron levels and neurological deficits arise prematurely in the tau^-/-^ mice. Indeed we previously did not observe further behavioral decline post 12 months in tau^-/-^ mice [5]. Recent data from our group (Lei et al. unpublished) indicate that by 24 months of age, the iron levels in normal mice catch up to the elevated iron levels in the tau^-/-^ mice, which reach a ceiling value. Taken together, these data characterize tau^-/-^ mice as an accelerated model for brain aging.

## Conclusion

Recent large-scale phase III clinical trials of drugs directly targeting the Aβ-related pathways involved in AD have failed to benefit patients [47,48], and therapeutic approaches targeting tau are gaining attention because reducing tau can ameliorate Aβ-induced toxicity [11,15,49,50] and rescue the toxicity in mouse mutant tau transgenic models [51-54]. Also, tau reduction is protective against epilepsy [6,49,55]. Enthusiasm for tau reduction as a therapeutic target is tempered by findings that complete tau ablation causes iron-mediated neurodegeneration with aging, which is, however, ameliorated by iron chelation [5] or antioxidant treatment [21]. In this study, we re-validated that excess reduction of tau protein leads to motor and cognitive consequences. Others have shown that tau protein has synaptic functions, and that tau ablation in aged mice or rats causes impaired LTD [17] and LTP [16]. Furthermore, one report warns that tau ablation worsens Aβ-induced cognitive impairment at an older age [56]. Taken together, tau may have important functions that sustain the longevity of neurons. Therefore, rather than targeting tau expression directly, microtubule stabilizers that restore tau function [57-59] or strategies that selectively lower iron content in the disease-affected brain [5,60-62], may be safer therapeutic approaches.

## Methods

### Mice

All mice were housed in a specific pathogen free (SPF) facility according to standard animal care protocols and fed standard laboratory chow (Code 102108, Barastoc, Ridley AgriProducts) and tap water *ad libitum*. All animal procedures were approved by the Florey Institute animal ethics committee (10-017-MHRI) and were performed in accordance with the National Health and Medical Research Council guidelines. Mice on a background of sv129 and C57Bl6 were originally obtained from Dr. M. Vitek (Duke University) [13] and were inbred before we set up a breeding strategy to back cross them for 10 generations onto a Bl6 background. Mice with both genetic backgrounds were maintained homozygously and mutants were backcrossed to the parental inbred strain every 3 generations. Gender distributions of the mice used in each experiment are given in Additional file 6: Table S1.

### L-DOPA treatment

Freshly dissolved L-DOPA in 0.9% NaCl, 0.5% Na-carboxymethylcellulose, 0.5% benzyl alcohol, 0.4% Tween-80, was administered by oral gavage useing of a blunted oral-esophageal needle (18 g, cropping needle) at 10 mg kg^-1^ for one dose. The performance tests were carried out 1 hour after the dose to allow maximal absorption.

### Accelerated Rotarod test

Accelerated Rotarod test was performed as previously described [5]. Briefly, mice were assessed on three occasions (separated by 1 hour interval) using a Panlab Rotarod apparatus in an accelerating paradigm (4 to 40 rpm, maximum time of 3 min; speed increases every 8 s). The time on the rod was recorded and the triplicates averaged for analysis. Trials were excluded if the mouse jumped off the rod.

### Pole test

Pole test was performed as previously described [5]. Briefly, mice were placed vertically facing upward on the top of a 30 cm vertical, 1cm diameter pole. The animals were habituated to the pole on the day before the trial by performing 5 consecutive mock trials. The trials on the day of testing were recorded by digital video for timing analysis. The length of time each mouse took to turn toward the ground (time to turn), and to reach the ground (time to finish) was recorded for each trial. Each mouse underwent five trials and the best trial was used in analysis. Trials were excluded if the mouse jumped or slid down the pole. The pole was cleaned between tests using 70% ethanol.

### Hindlimb Clasping test

Method for Hindlimb clasping test was adapted from Lieu et al. [24]. Briefly, mice were suspended by the base of the tail and their behaviors were recorded for 30 seconds. Three separate trials were taken over course of test (1 hour apart). Hindlimb clasping was rated from 0 to 3 based on severity: 0 = hindlimbs splayed outward and away from the abdomen, 1 = one hindlimb retracted inwards towards the abdomen for at least 50% of the observation period, 2 = both hindlimbs partially retracted inwards towards the abdomen for at least 50% of the observation period, 3 = both hindlimbs completely retracted inwards towards the abdomen for at least 50% of the observation period. Scores of 0.5 were used when appropriate. Hindlimb clasping severity scores were calculated by averaging the three separate tests.

### Open Field test

Open Field test was performed as previously described [5]. Briefly, spontaneous motor activity of mice in an enclosed arena (27.3 cm wide, 27.3 cm deep, 20.3 cm high) was monitored using a 48 Channel IR Controller photo-beam activity system (Med Associates Inc, USA) and analyzed by Activity Monitor (Version 6.02, Med Associates Inc, USA). The mouse was placed into the chamber for one hour, and the data analyzed in 5 min time bins. Movement parameters were calculated from the interception of beams that provided XY coordinates.

### Digigait test

The ambulatory gait analysis of mice was quantified using the DigiGait^TM^ Imaging System (Mouse Specifics Inc., USA). A video camera mounted below a motorized transparent treadmill belt captures the ventral side of each mouse while walking. The paws of the mice were colored with a red marker to improve contrast for automated analysis. Mice were placed on the treadmill and rapidly accelerated to 15 cm/sec at which speed data was captured for analysis. The total time of recording was approximately one minute. Numerous postural and kinematic metrics of gait dynamics were determined by Digigait 8 (Mouse Specifics Inc., USA), representing the temporal record of paw placement relative to the treadmill belt.

### Y maze test

Y maze test was performed as previously described [5]. Briefly, a Y-shaped grey-painted timber with arms (29.5 cm long × 7.5 cm wide × 15.5 cm high) was used to assess spatial recognition memory. All mice underwent a 2-part Y-maze test separated by a 1 h interval. The three identical arms were randomly assigned start arm, novel arm, and other arm. Visual cues were placed on the walls of the maze. In the first part of the test (training), the novel arm was occluded and mice were placed in the start section of the maze and were allowed to freely explore for 10 min. For the second part (test), access to the novel arm was made available and mice were placed back in the maze in the same starting arm, and allowed to explore for 5 min. Behaviors were recorded on video during a 5 min trial and the TopScan Realtime Option Version 2.00 (Clever Sys Inc, USA) was used for analysis. Data are expressed as the percentage of frequency and duration for novel arm entries made during the 5-min second trial. All testing performed during the light phase of the circadian cycle.

### Mouse brain preparation

Mouse brain samples were prepared as previously described [5]. Briefly, mice were euthanized with an overdose of sodium pentobarbitone (Lethabard, 100mg/kg) and perfused with ice-cold saline. The left brain hemisphere was fixed in 4% paraformaldehyde for 24 h, and then transferred to 30% Sucrose + PBS (pH 7.4) and kept at 4°C overnight for tyrosine hydroxylase (TH) immunohistochemistry.

### Stereological estimation of SN TH+ neurons

Tyrosine hydroxylase immunohistochemistry and SN neuron counting were performed as previously described [5]. Briefly, brains were frozen sectioned on a calibrated Leica Cryostat in 30 μm sections. Sections (1:3 series) were collected through the SN pars compacta (SNpc) (anteroposterior -2.92 to -3.64 mm from bregma). After brief fixation (4% paraformaldehyde for 30 seconds), the sections were blocked in 3% normal goat serum (Millipore) and incubated with primary anti-TH rabbit polyclonal (1:3000, Millipore) overnight. The sections were then incubated with goat anti-rabbit secondary HRP-conjugated antibody for 3 hours (Millipore), followed by diaminobenzidine solution (1% in PBS + 1% CoCl_2_, 1% NiSO_4_) + 3% hydrogen peroxide (1:3000). The numbers of neurons within the SNpc were then estimated using a stereological fractionator design. The counts were taken using an unbiased counting frame of x = 35 μm, y = 45 μm (1575 μm^2^) at regular intervals on a sampling grid of x = 140 μm, y = 140 μm (19600 μm^2^), viewed with a 60 x 1.3 N.A. oil objective (DMLB Leica Microscope) by the morphometry and design-based stereology software package (Stereo Investigator 10.04, Microbrightfield, Colchester, VT).

### Metal measurement

Samples were prepared as previously described [5]. Briefly, the mouse chow was re-suspended in 65% nitric acid (Merck) overnight. The samples were then heated for 20 min at 90°C. The samples were diluted in double-distilled water and assayed by an inductively coupled plasma mass spectrometer (Agilent 7700). Each sample was measured in triplicate and the concentrations determined from the standard curve were normalized to weight.

### Statistics

Statistical analysis was carried out in Prism 6 (GraphPad Software Inc). All tests were two-tailed, with the level of significance set at 0.05. Detailed tests used in each experiment are described in Figure legends.

## Competing interests

Dr Finkelstein is a shareholder in and paid scientific consultants for Prana Biotechnology Pty Ltd. Dr. Bush is a shareholder in Prana Biotechnology Pty Ltd., Eucalyptus Pty Ltd., Mesoblast Pty Ltd. and a paid consultant for Collaborative Medicinal Developments LLC and Brighton Biotech LLC.

## Authors’ contributions

Scientific concept: PL, AIB. Experimental design: PL, SA, DIF, AIB. Experiments: PL, SA, SM, QZ, IV. Manuscript preparation: PL, AIB. Manuscript edit: all authors. All authors read and approved the final manuscript.

## Supplementary Material

Additional file 1: Figure S1. No correlation between weight and Rotarod test performance in 12-month-old mice. Click here for file

Additional file 2: Figure S2. No correlation between weight and Pole test performance in 12-month-old mice. Click here for file

Additional file 3: Figure S3. Temporal Open field profile. Click here for file

Additional file 4: Figure S4. Analysis on 10 mins movement in Open field test. Click here for file

Additional file 5: Figure S5. Additional parameters analyzed by Digigait apparatus. Click here for file

Additional file 6: Table S1. Mouse number and gender used in each experiment. Click here for file
