# Supplementary material for: Motor and cognitive deficits in aged tau knockout mice in two background strains
Source: Mol Neurodegener. 2014 Aug 14;9:29. doi: 10.1186/1750-1326-9-29 (PMC4141346; doi:10.1186/1750-1326-9-29)
Supplement: Additional file 1: Figure S1. — No correlation between weight and Rotarod test performance in 12-month-old mice. [file 1750-1326-9-29-S1.pdf]

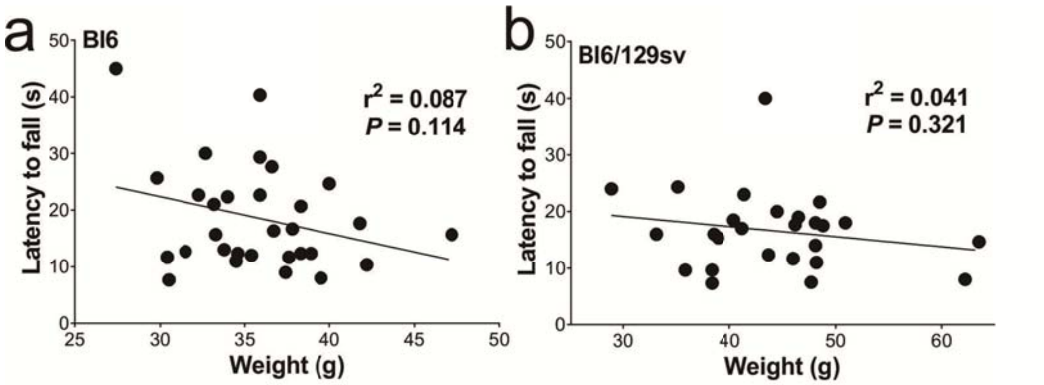

Figure S1. No correlation between weight and Rotarod test performance in 12-month-old mice (n as indicated in **Figure 2**). **a**) Between all mice in Bl6 background and weight (r^2 = 0.087, p = 0.114). **b**) Between all mice in Bl6/129sv background and weight (r^2 = 0.041, p = 0.321).
