# Supplementary material for: Motor and cognitive deficits in aged tau knockout mice in two background strains
Source: Mol Neurodegener. 2014 Aug 14;9:29. doi: 10.1186/1750-1326-9-29 (PMC4141346; doi:10.1186/1750-1326-9-29)
Supplement: Additional file 2: Figure S2. — No correlation between weight and Pole test performance in 12-month-old mice. [file 1750-1326-9-29-S2.pdf]

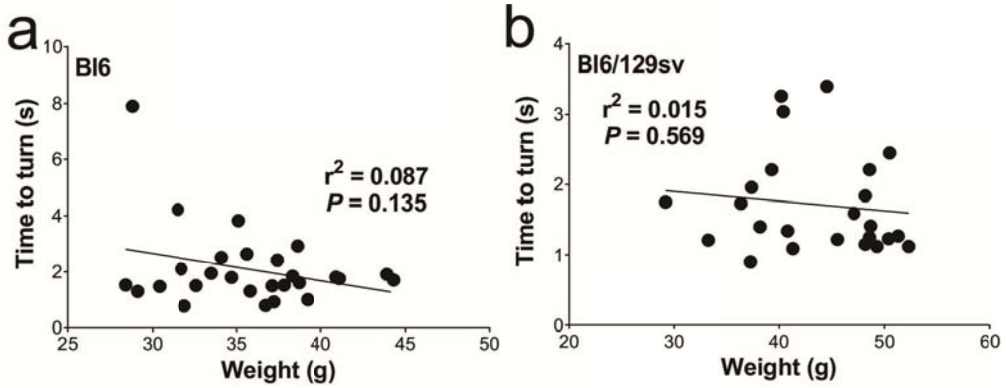

Figure S2. No correlation between weight and Pole test performance in 12-month-old mice (n as indicated in **Figure 3**). **a**) Between all mice in Bl6 background and weight ($r^2 = 0.087$, $p = 0.135$ by linear regression). **c**) Between all mice in Bl6/129sv background and weight ($r^2 = 0.015$, $p = 0.569$).
