# Supplementary material for: Motor and cognitive deficits in aged tau knockout mice in two background strains
Source: Mol Neurodegener. 2014 Aug 14;9:29. doi: 10.1186/1750-1326-9-29 (PMC4141346; doi:10.1186/1750-1326-9-29)
Supplement: Additional file 3: Figure S3. — Temporal Open field profile. [file 1750-1326-9-29-S3.pdf]

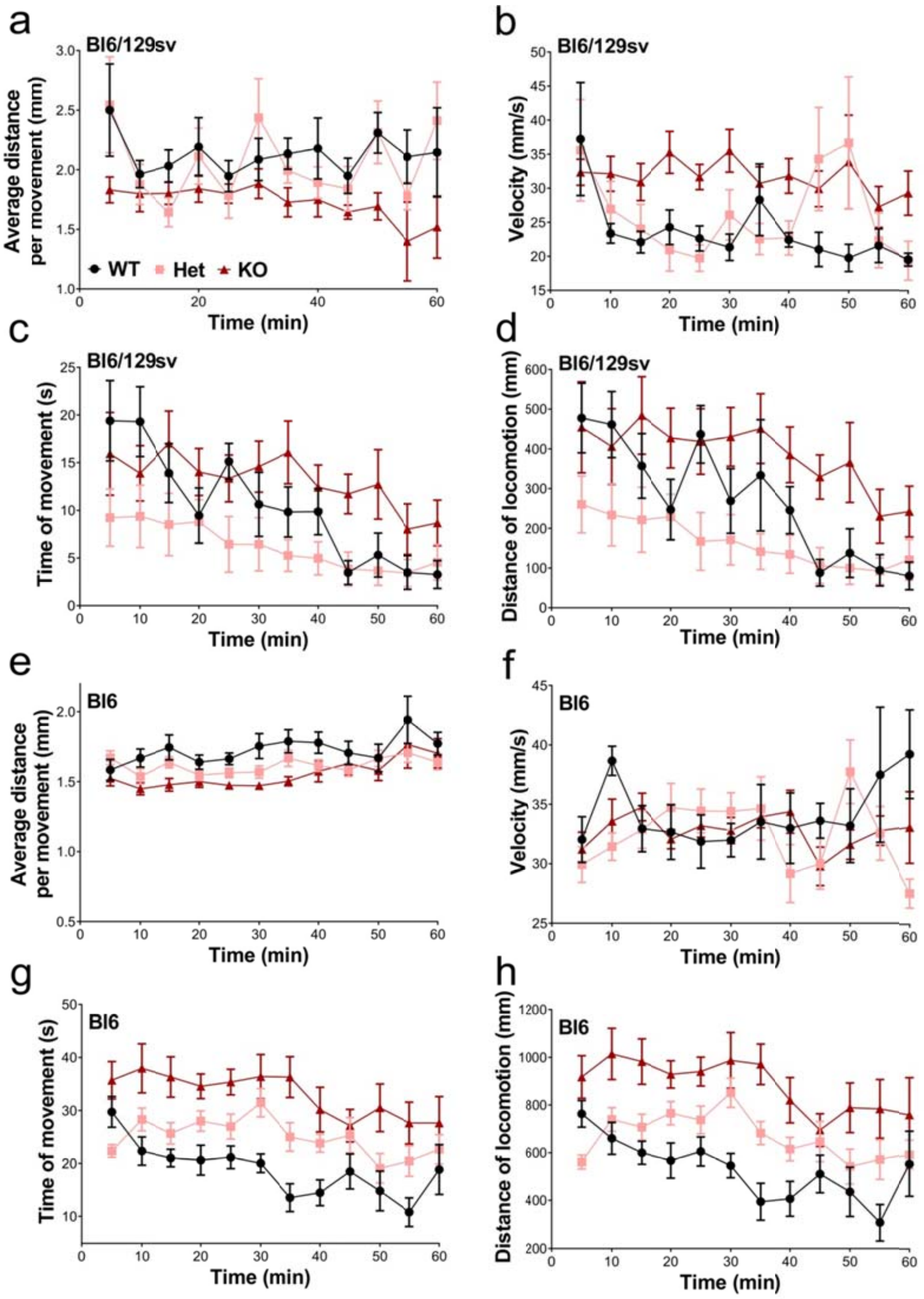

Figure S3. Temporal Open field profile. **a-d**) Temporal profile of mice in Bl6 background during the Open field test. **a**) Average distance per movement. **b)** Velocity. **c)** Time of movement. **d)** Distance of locomotion. **e-g**) Temporal profile of mice in Bl6/129sv background during the Open field test. **e**) Average distance per movement. **f)** Velocity. **g)** Time of movement. **h)** Distance of locomotion. n as indicated in **Figure 4**. Data are means ± SEM.
