# Supplementary material for: Motor and cognitive deficits in aged tau knockout mice in two background strains
Source: Mol Neurodegener. 2014 Aug 14;9:29. doi: 10.1186/1750-1326-9-29 (PMC4141346; doi:10.1186/1750-1326-9-29)
Supplement: Additional file 4: Figure S4. — Analysis on 10 mins movement in Open field test. [file 1750-1326-9-29-S4.pdf]

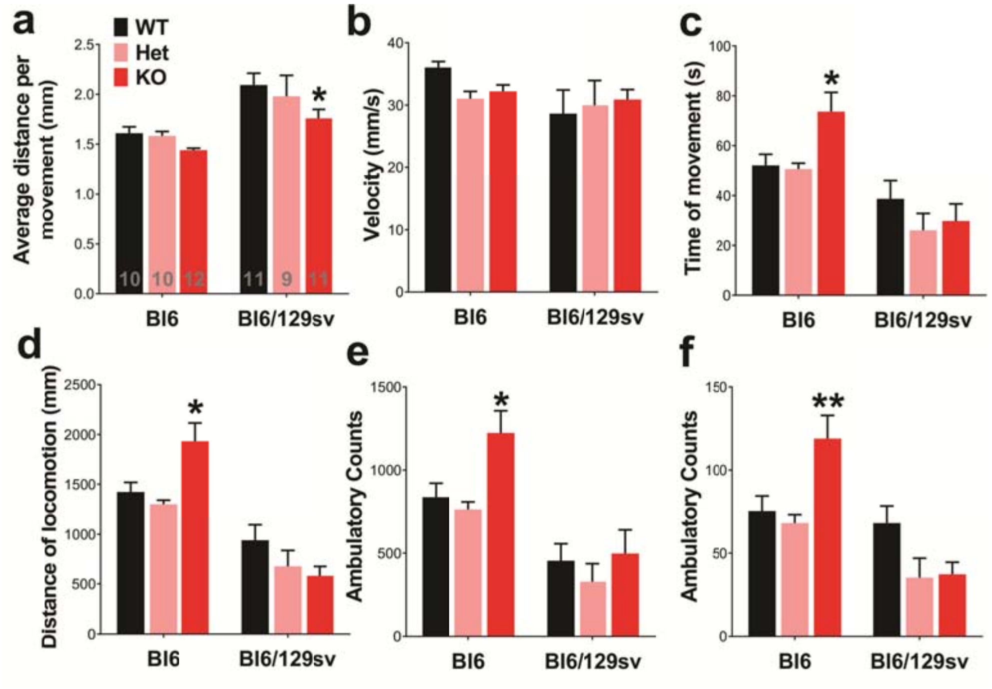

Figure S4. Analysis of movement during first 10mins in Open field. **a)** Average distance per movement. Two-way ANOVA: genotype (p = 0.0155) and genetic background (p < 0.0001) effects but no interaction (p = 0.636). **b)** Velocity. Two-way ANOVA: no genetic background (p = 0.09), or genotype effects (p = 0.749), or interaction (p = 0.318). **c)** Time of movement. Two-way ANOVA: genetic background (p < 0.0001) effects, but no genotype effect (p = 0.1375) or interaction (p = 0.054). **d)** Distance of locomotion. Two-way ANOVA: genetic background effect (p < 0.0001) and interaction (p = 0.005), but no genotype effect (p = 0.1707). **e)** Ambulatory counts of movement in the central field. Two-way ANOVA: genotype (p < 0.022) and genetic background (p < 0.0001) effects, but no interaction (p = 0.244). **f)** Ambulatory counts of movement in the peripheral field. Two-way ANOVA: genotype (p = 0.035) and genetic background (p < 0.0001) effects, plus interaction (p = 0.002). * p < 0.05, ** p < 0.01, *** p < 0.001, versus age-matched wild type mice (Bonferroni post hoc test). n as indicated. Data are means ± SEM.
