# Supplementary material for: Motor and cognitive deficits in aged tau knockout mice in two background strains
Source: Mol Neurodegener. 2014 Aug 14;9:29. doi: 10.1186/1750-1326-9-29 (PMC4141346; doi:10.1186/1750-1326-9-29)
Supplement: Additional file 5: Figure S5. — Additional parameters analyzed by Digigait apparatus. [file 1750-1326-9-29-S5.pdf]

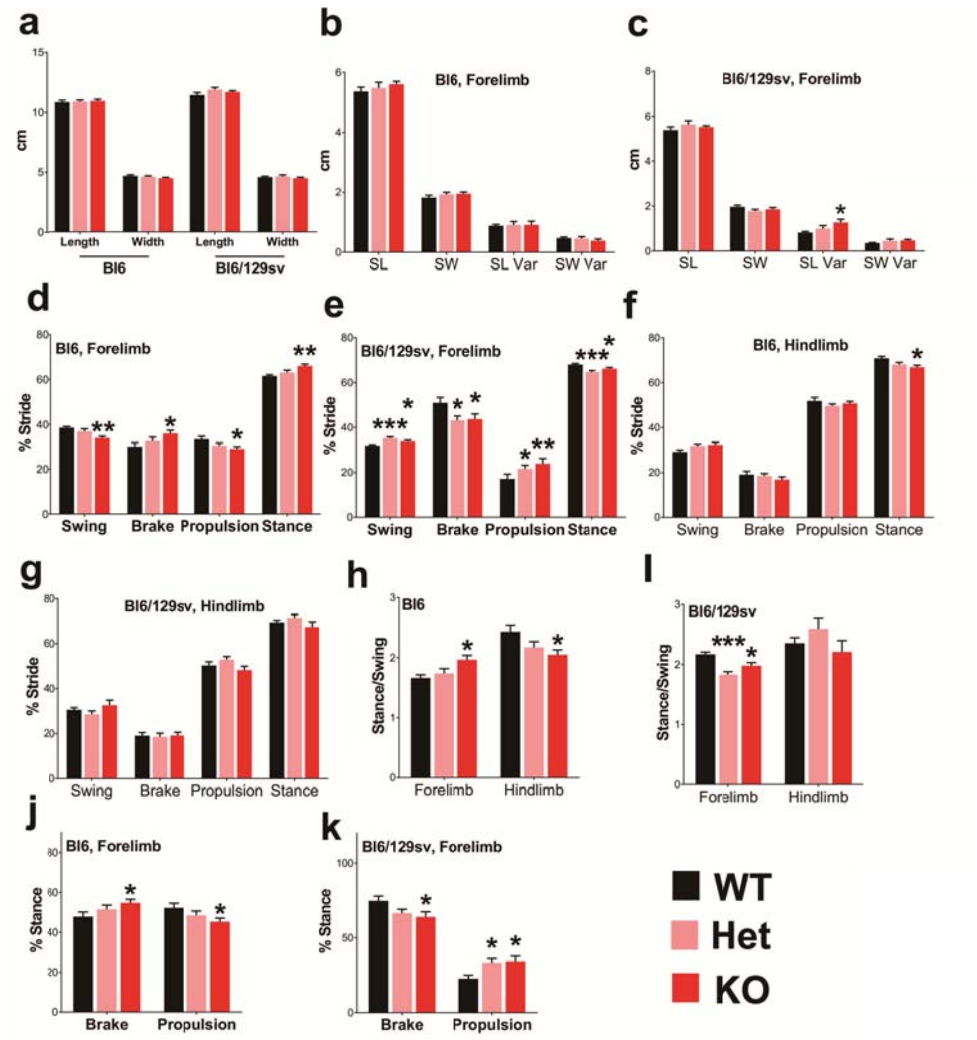

Figure S5. Additional parameters analyzed by Digigait apparatus. **a-k**) parameters from Digigait analysis: the length and width of mice (**a**), stride length, stance width, and variability of forelimb stride of mice in Bl6 background (**b**), stride length, stance width, and variability of forelimb stride of mice in Bl6/129sv background (**c**), mean percent time spent in the swing, brake, propulsion, and stance phase of stride for forelimbs of mice in Bl6 background (**d**), mean percent time spent in the swing, brake, propulsion, and stance phase of stride for forelimbs of mice in Bl6/129sv background (**e**), mean percent time spent in the swing, brake, propulsion, and stance phase of stride for hindlimbs of mice in Bl6 background (**f**), mean percent time spent in the swing, brake, propulsion, and stance phase of stride for hindlimbs of mice in Bl6/129sv background (**g**), the ration between stance and swing for mice in Bl6 background (**h**), the ratio between stance and swing for mice in Bl6/129sv background (**i**), mean percent time spent in the brake and propulsion phase of stance for forelimbs of mice in Bl6 background (**j**), and mean percent time spent in the brake and propulsion phase of stance for forelimbs of mice in Bl6/129sv background (**k**) One-way ANOVAs were performed to test the differences versus age-matched wild type mice for each individual parameter. * $p < 0.05$, ** $p < 0.01$, *** $p < 0.001$**.** n as indicated. Data are means ± SEM.
