# Supplementary material for: Motor and cognitive deficits in aged tau knockout mice in two background strains
Source: Mol Neurodegener. 2014 Aug 14;9:29. doi: 10.1186/1750-1326-9-29 (PMC4141346; doi:10.1186/1750-1326-9-29)
Supplement: Additional file 6: Table S1. — Mouse number and gender used in each experiment. [file 1750-1326-9-29-S6.pdf]

Table S1: Mouse number and gender used in each experiment.

	Bl6			Bl6/129sv		
**Figure number**	**WT**	**Het**	**KO**	**WT**	**Het**	**KO**
**1**	22 (10F)	13 (5F)	18 (9F)	26 (16F)	15 (8F)	26 (13F)
**2-6, S1-5**	10 (1F)	10 (8F)	12 (7F)	11 (5F)	9 (6F)	11 (4F)
**7a-d**	Sham 10 (4F); L-DOPA 11 (7F)	N/A	Sham 9 (6F); L-DOPA 10 (2F)	Sham 10 (6F); L-DOPA 10 (5F)	N/A	Sham 10 (5F); L-DOPA 10 (8F)
**7e**	5 (1F)	5 (3F)	5 (2F)	5 (3F)	5 (3F)	5 (2F)
**8**	22 (7F)	10 (8F)	22 (13F)	11 (5F)	9 (6F)	11 (4F)
